# Conference Report: SYMPOSIUM ON OPTICAL FIBER MEASUREMENTS Boulder, CO September 15–17, 1992

**DOI:** 10.6028/jres.098.021

**Published:** 1993

**Authors:** Douglas L. Franzen, Gordon W. Day

**Affiliations:** Electromagnetic Technology Division, National Institute of Standards and Technology, Boulder, CO 80303

The seventh Symposium on Optical Fiber Measurements was held on September 15–17, 1992 at NIST in Boulder, CO. Over 220 people attended the two and a half day meeting and heard a program consisting of 11 invited and 43 contributed papers with over half the papers coming from outside the United States. In addition, 10 committees associated with standards organizations like the Telecommunication Industry Association (TIA) and International Electrotechnical Commission (IEC) met in conjunction with the Symposium. The meeting covered metrology aspects of lightwave communications. While optical fiber measurements were emphasized, other lightwave components were also addressed including sources, detectors, integrated optic advices, passive filters, splitters, and connectors. The meeting scope covered metrology in both laboratory and field environments.

Important technical topics of the meeting included optical time domain reflectometry (OTDR),single-mode fiber geometry, polarization effects in fiber, and optical fiber amplifiers. OTDRs have unique measurement capabilities and represent the largest market segment of fiber optic test equipment. They therefore continue to be a topic of interest at the Symposia. T. Horiguchi and other workers from Nippon Telegraph and Telephone (NTT) reported on an OTDR operating at 1600 nm. Usual OTDRs operate at typical system wavelengths of either 1300 or 1550 nm. By operating at longer wavelengths, where single-mode fiber bending loss is higher, the new OTDR has improved sensitivity for locating potential faults caused by excessive bending. Operation at 1600 nm is accomplished by using a Q-switched erbium fiber laser to pump a fiber Raman laser. A dynamic range of 24.5 dB is achieved with a resolution of 20 m.

Future fiber networks may contain several fiber splitters for distribution. F. Kapron and J. Berardinelli from Bellcore described an algorithm for determining branch fault loss and reflectance when the OTDR “looks” through a splitter. To better quantify OTDR signatures, A. Judy from AT&T Bell Laboratories developed an expression which relates fiber backscatter and reflectance. J. Clayton and others from AT&T Bell Laboratories performed statistical analysis to determine the limitations of OTDRs when predicting fiber uniformity.

Low coherence reflectometry is closely related in function to OTDR. It is an interferometric technique for obtaining very high resolution on short lengths of fiber. K. Takada from NTT reviewed the method in an invited paper. W. Sorin and D. Baney of Hewlett Packard described a method for improving the accuracy of reflectance measurements in the presence of fiber chromatic dispersion.

Geometrical dimensions of single-mode fiber continue to be an important issue. Low loss splices and connectors depend on dimensional integrity. The common measurement method of near-field video analysis requires a standard for absolute calibration. In an invited paper, J. Baines and K. Raine of the National Physical Laboratory (NPL) reviewed recent developments in fiber geometry measurements and concluded standards laboratories need to determine cladding diameter with uncertainties of 0.1 µm (nominal cladding diameter is 125 µm). Measurement methods in use at NPL include a Michelson white light interferometer, an image inverting microscope, and a scanning confocal microscope.

M. Young, P. Hale, and S. Mechels described geometry measurements at NIST where a contact micrometer, scanning confocal microscope, and white light interference microscope are used to determine cladding diameter. They estimate overall uncertainty for the three methods as 50, 40, and 30 nm, respectively. Of the three methods, the micrometer is most efficient to use; therefore, it will be the method employed by NIST in dispensing standard reference fibers. In a separate paper, P. Hale of NIST described the interference microscope in more detail. The partially automated system is based on a Mirau interference objective and a specially designed fixture for holding the fiber on an optical flat. A comparison of the micrometer and confocal microscope to the interference microscope, on eight fiber diameters, yielded average rms differences of 15 and 29 nm.

The geometry of the plastic buffer coatings surrounding the fiber is also important. Buffer coatings protect the fiber from the environment and should be concentric to provide good centering in ribbon cable. A. Hallam and J. Shaw described a conventional gray scale near-field system which is modified to measure coating thickness and concentricity. The modification is an oil filled cell which uses dark-field illumination and views the fiber from the side. Typical repeatabilities for coating diameter and concentricity are 0.2 and 0.25 µm. Hermetic fiber coatings of thinly deposited carbon (tens of nanometers) are frequently used to improve fiber strength and suppress hydrogen induced loss. Y. Ishida and others from Furukawa Electric make use of the electrical properties of the carbon to measure the coatings during draw. By measuring the complex impedance, they are able to measure coating thickness with a resolution of ± 1 nm for thicknesses over 17 nm.

Polarization effects in fiber can be useful for sensor applications; however, they can also be detrimental to communications systems where polarization mode dispersion (PMD) reduces system bandwidth. In an invited paper, B. Hefner of Hewlett Packard described a polarimeter and summarized the progress in polarization measurements. Y. Gu and L. Poyntz-Wright from York Technologies discussed methods for measuring extinction ratios in polarization-maintaining fiber, while L. Stokes of Hewlett Packard presented accurate measurements of polarization dependent loss of fiber components as a function of wavelength. Unwanted polarization mode dispersion in conventional fiber arises from core deformation and external stress. While the effects are small in kilometer lengths, fibers in future undersea systems using erbium fiber amplifiers are likely to be hundreds of kilometers long, so distortion could result at high bit rates. Y. Namihira and J. Maeda from KDD of Japan compared several methods for measuring PMD, and concluded that the interfero-metric method is best for measuring small PMD values. L. Thevenaz, M. Nikles, and P. Robert from the Swiss Federal Institute of Technology described a novel PMD measurement method based on an interferometric loop of fiber. The method is very easy to implement in the laboratory and gives the probability distribution of the PMD.

Five papers were concerned with optical fiber amplifiers. This new technology promises to revolutionize long fiber systems. D. Hall of Corning, in an invited paper, discussed the characterization of erbium fiber amplifiers. Measurements requiring immediate attention include gain, noise, and distortion. Reflections within the measurement system are potential sources of error in gain measurements. Gain measurements at specific wavelengths in the presence of power at other wavelengths have not been addressed; those types of measurements will be necessary for wavelength-division-multiplexed systems. Analog cable TV signals require low distortion; measurements need to be developed to characterize erbium fiber amplifiers for this requirement. R. Hickernell and others from NTT reported on chromatic dispersion measurements in erbium doped fiber. By using techniques of Fourier transform spectroscopy, they concluded the dispersion resulting from resonant gain or loss can be significant compared to background fiber dispersion. M. Artiglia and other CSELT of Italy colleagues addressed measurements of noise figure. They concluded that excessnoise from the source can affect the results; a procedure to correct data for excess noise is given. T. Kashiwada and others from Sumitomo evaluated potential sources of error in noise figure measurement; improved results were obtained through the use of a bandpass filter and a method of polarization control. There is a significant market for erbium fiber amplifiers. Automated measurements are therefore cost effective. D. Cole and M. Vance from Corning evaluated an automated system for measuring gain, output power, amplified spontaneous emission (ASE), and unabsorbed pump power as a function of input power and wavelength.

Four page summaries of papers given at the Symposium are contained in a 245 page meeting digest. The *Technical Digest—Symposium on Optical Fiber Measurements, 1992* is available as NIST Special Publication 839. A copy can be obtained by contacting either D. L. Franzen (303) 497-3346 or G. W. Day (303) 497-5204; copies are free of charge as long as supplies last. Alternatively, the Digest can be purchased from the Superintendent of Documents, U.S. Government Printing Office, Washington, DC 20402 or the National Technical Information Service (NTIS), Springfield, VA 22161.

